# Small-Sized Clone of T Cells in Multiple Myeloma Patient after Auto-SCT: T-LGL Leukemia Type or Clonal T-Cell Aberration?

**DOI:** 10.1155/2013/417353

**Published:** 2013-04-21

**Authors:** Giuseppe Mele, Marilena Greco, Maria Rosaria Coppi, Giacomo Loseto, Angela Melpignano, Salvatore Mauro, Gianni Quarta

**Affiliations:** ^1^Haematology Division and BMT Unit, Antonio Perrino Hospital, 72100 Brindisi, Italy; ^2^Laboratory of Genetic, Vito Fazzi Hospital, 73100 Lecce, Italy

## Abstract

Second cancers and particularly postransplant lymphoproliferative disorders (PTLDs) are extremely rare in patients undergoing autologous peripheral blood stem cell transplantation (auto-SCT). We report the case of clonally rearranged T-cell expansion which occurred after auto-SCT for Multiple Myeloma (MM). Does asymptomatic clonal T-cell large granular lymphocytic proliferation, in our experience, represent either a secondary cancer after auto-SCT or clonal T cell aberration or derive from expansion of coexisting undetected small-sized clone of T cells?

## 1. Introduction 

PTLDs were initially recognized in solid organ transplant recipients, and their incidence can range from 1% to 6%. Among patients undergoing haematopoietic cell transplantation, PTLDs occur almost exclusively in recipients of allogeneic grafts (allo-SCT) with an overall incidence rate from 1% to 2% and manifest early within the first year from transplantation [1–3]. Second cancers and particularly PTLDs are extremely rare in patients undergoing auto-SCT [4–7]; according to Fenk et al., the median time from diagnosis of MM to the occurrence of secondary malignancies is 56 months [7]. The majority of PTLDs are of B-cell origin but they may also originate from T cells [8], but very rarely from natural killer cells [9].

We report the case of clonally rearranged T-cell expansion mimicking T-cell large granular lymphocytic (T-LGL) leukemia which occurred after auto-SCT for MM. The diagnosis of T-LGL leukemia requires a multiparametric approach including peripheral blood examination, bone marrow aspirate and bone marrow trephine biopsy with immunohistochemistry, flow cytometric immunophenotyping, and molecular analysis for TCR gene rearrangements. According to the World Health Organization classification of lymphoid neoplasms, T-LGL leukemia is characterized by a persistent (>3 months) increase of large granular lymphocyte (2 × 10^9^/L); cases with an LGL count of <2 × 10^9^/L can be diagnosed as T-LGL leukemia only if clonal rearrangement of T-cell receptor (TCR) gene is demonstrated. T-LGL leukemia is distinguished from reactive polyclonal granular lymphocytes through both the aberrant coexpression of the natural killer cell-associated antigen CD57 and demonstration of T-cell clonality.

## 2. Case Report

**Figure 1:**
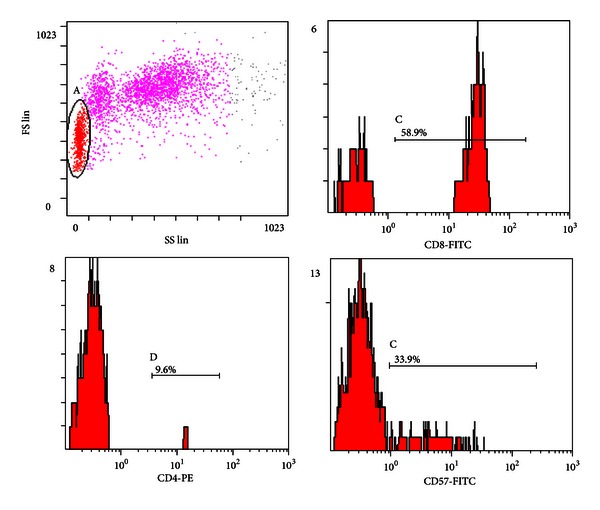
Immunophenotypic features of T-cell clonal lymphoproliferative disorder (CD4−, CD8+, CD57+).

**Figure 2:**
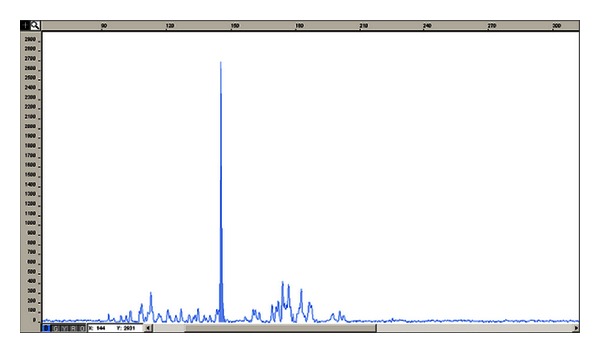
Monoclonal T population evaluated after PCR amplification of the VJ rearranged segments of the TCR-*γ* gene.

 A 70-year-old male patient, undergoing a first auto-SCT for stage IIIA MM IgA*λ*, manifested early an asymptomatic clinically lymphoproliferative disorder similar to T-LGL leukemia. He had been conditioned with melphalan 140 mg/m^2^ after induction therapy with VTD regimen (Bortezomib 1,3 mg/m^2^ on days 1, 4, 8, 11; Thalidomide 100 mg/d; Dexamethasone 40 mg once daily i.v. on days 1→2, 4→5, 8→9, 11→12, every 28 days) because of chronic obstructive bronchopneumonia and advanced age. On day 0, he received cryopreserved peripheral blood stem cell (4 × 10^6^ CD34+ cells/kg). Engraftment was prompt, neutrophil recovery >0,5 × 10^9^/L and platelets >20 × 10^9^/L occurring on days +11 and +13, respectively. On day +30 and +60 in serum and urine M protein was undetectable by immunofixation and on electrophoresis. Bone marrow aspirate with flow cytometric immunophenotyping and bone marrow trephine biopsy with immunohistochemistry confirmed the absence of clonal plasma cells. Surprisingly, on day +43 from auto-SCT, peripheral blood smear showed absolute lymphocytosis (WBC count 14.9 × 10^9^ cells/L; absolute lymphocytic count 9.5 × 10^9^ cells/L). The bone marrow aspirate and bone marrow trephine biopsy confirmed the presence of a population of large granular lymphocytes, mimicking a chronic lymphoproliferative disorder. Immunohistochemistry and flow cytometry immunophenotyping, performed on fresh cells obtained from both bone marrow and peripheral blood, showed T-cell-associated antigens (CD3+CD4−CD8+CD56−CD57+) (Figure 1). Serological tests for hepatitis C and B virus, CMV, EBV, Parvovirus B19, Toxoplasma gondii, and HIV were negative. Monoclonal rearrangement of TCR-*γ* by PCR was detected in the peripheral blood and bone marrow (Figure 2); heavy immunoglobulin and light chain genes were not rearranged. On FDG-TC/PET, no lymphoadenopathy and splenomegaly were observed. On day +111 from auto-SCT, although there was a sharp reduction in the number of lymphocytes (WBC count 7.4 × 10^9^ cells/L with 45% lymphocytes; absolute lymphocytic count 3.3 × 10^9^ cells/L), immunophenotypic study was similar to the previous one. Therefore, laboratory results and long observation time suggested the diagnosis of T-cell clonal lymphoproliferative disorder similar to T-LGL leukemia. The patient remained in stringent complete remission from MM, according to the International Myeloma Working Group (IMWG) criteria, more than 6 months after auto-SCT. On day +243 from auto-SCT, the patient presented with weight loss, asthenia, diffuse bone pain, and slight persistent fever. Serum protein electrophoresis showed hypergammaglobulinemia with evidence of a monoclonal spike (1,5 gr/dL). In the bone marrow, an extensive infiltration of the immature plasma cells together with very small T-cell population CD3+CD4−CD8+CD56−CD57+, clonally rearranged, was observed. Salvage therapy with Bortezomib 1,3 mg/m^2^ on days 1, 4, 8, and 11 and Dexamethasone 20 mg once daily on days 1→2, 4→5, 8→9, 11→12, every 21 days, was given. After two cycles, the patient developed severe deterioration of his clinical conditions. A significant increase of M-spike (3,4 gr/dL) was seen. A therapy with Bendamustine (80 mg/m^2^ on days 1, 2) + Dexamethasone (20 mg once daily i.v. on days 1→4 and 15→18) and Lenalidomide (10 mg daily on days 1→21), every 28 days, outside of clinical trials in a compassionate use program, was started as a final attempt. Very good partial remission according to IMWG criteria was observed after only two cycles of chemotherapy; after the fourth courses, the M-spike disappeared completely. Immunophenotypic analysis and monoclonal rearrangement study, performed on fresh cells obtained from both bone marrow and peripheral blood, confirmed the absence of clonal plasma cells but showed the persistence of small-sized population CD3+CD8+CD57+, clonally rearranged (WBC count 2.9 × 10^9^ cells/L; absolute lymphocytic count 0.3 × 10^9^ cells/L).

## 3. Results and Discussion 

This case is of particular interest because of the surprising persistence of a very small clonal T-LGL population. Several pathogenetic mechanisms and heterogeneous clinical scenarios can be hypothesized. (1) T-LGL leukemia has been seen after solid organ transplantation and allo-SCT and typically manifests within the first year [1–3]. The PTLDs and particularly T-LGL leukemia are extremely rare in auto-SCT patients [4–7]. In allo/auto-SCT, EBV serologic status, CMV reactivations, and viral infections may represent a pathologic state in which chronic antigenic stimulation may result in T-cell clonal expansions, although recent studies were unable to demonstrate a proved relationship cause-effect between T-LGL leukemia and CMV infection, because CMV reactivation represents very frequent event post-CST and although de novo T-LGL leukemia is not associated with EBV [10]. Intensity of immunosuppression and additional genotoxic stress of therapeutic programs may represent further risk factors, although auto-SCT is a procedure less immunosuppressive than allo-SCT. (2) In MM about one-third of patients may show monoclonal TCR-*β* rearrangements by Southern blot of the peripheral blood and expanded T-cell populations may share an immunophenotype similar to T-LGL leukemia (CD3+CD8+CD57+). (3) Finally, there are examples of T-LGL leukemia concurrent with MM.

## 4. Conclusions

 Does *asymptomatic clonal LGL proliferation*, in our experience, represent either a secondary cancer after auto-SCT or clonal T-cell aberration or derive from expansion of coexisting undetected small-sized clone of T cells? An evident expanded T-cell population is absent at diagnosis; the time interval of occurrence of expanded T-cell population from auto-SCT is very short, and all viral serological tests are negative. So, in our opinion, the identification of the event sequence appears of particular difficulty.
